# Circulating tumour DNA as a predictor of survival of patients with diffuse large B‐cell lymphoma in a daily practice

**DOI:** 10.1111/bjh.70128

**Published:** 2025-09-07

**Authors:** Prokop Vodicka, Iva Hamova, Adriana Velasova, Kristyna Kupcova, Petra Zemankova, Petr Nehasil, Anton Tkachenko, Katerina Lochovska, Sarka Muzikova, Sarka Hrabetova, Jitka Dlouha, Petra Blahovcova, Tomas Frouz, Jana Senavova, Lucie Dlouha, Kamila Polgarova, Magdalena Klanova, Jana Salkova, Katerina Benesova, Pavel Klener, Marek Trneny, Ondrej Havranek

**Affiliations:** ^1^ First Department of Medicine‐Hematology, First Faculty of Medicine Charles University and General University Hospital Prague Czech Republic; ^2^ First Faculty of Medicine, BIOCEV Charles University Vestec Czech Republic; ^3^ First Faculty of Medicine, Institute of Medical Biochemistry and Laboratory Diagnostics, Charles University and General University Hospital in Prague Prague Czech Republic; ^4^ First Faculty of Medicine, Institute of Pathological Physiology Charles University Prague Czech Republic; ^5^ Department of Paediatrics and Inherited Metabolic Disorders, First Faculty of Medicine Charles University and General University Hospital in Prague Prague Czech Republic; ^6^ The Czech Lymphoma Study Group Prague Czech Republic

**Keywords:** circulating tumour DNA, diffuse large B‐cell lymphoma, minimal residual disease, risk stratification, treatment response evaluation

## Abstract

Circulating tumour DNA (ctDNA) is a promising biomarker for diffuse large B‐cell lymphoma (DLBCL) risk stratification and treatment response assessment, but real‐world studies were limited. Using a targeted sequencing approach (521‐gene panel), we showed that (1) baseline ctDNA level correlated with tumour burden and was an independent predictor of treatment outcome, (2) achievement of minimal residual disease (MRD) negativity was associated with a better treatment outcome and (3) interim MRD‐positivity combined with positron emission tomography/computed tomography scan‐positivity identified a high‐risk subgroup of DLBCL patients. Baseline ctDNA level and treatment related achievement of MRD negativity are valuable prognostic tools in DLBCL to improve risk stratification in routine clinical practice.

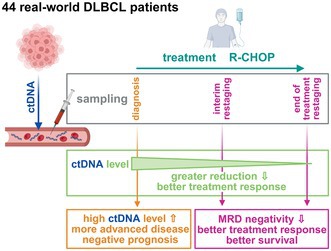

To the Editor,

Diffuse large B‐cell lymphoma (DLBCL) is a relatively well treatable disease with long‐term cure rates between 60% and 70% following standard front‐line immunochemotherapy R‐CHOP (i.e. rituximab, cyclophosphamide, doxorubicin, vincristine and prednisone) or Pola‐R‐CHP (polatuzumab vedotin, rituximab, cyclophosphamide, doxorubicin and prednisone).^1^, ^2^ Despite recent treatment advances, 30%–40% of DLBCL patients have primary refractory disease or experience a relapse; both associated with inferior survival outcomes. Importantly, none of the currently used prognostic tools can clearly identify these patients.^3^ Circulating tumour DNA (ctDNA) is a promising, non‐invasive biomarker that has demonstrated prognostic value across multiple cancer types, including DLBCL.^4^ Prior studies have shown that ctDNA levels at diagnosis correlate with key DLBCL characteristics, such as clinical stage, serum lactate dehydrogenase (LDH) levels and international prognostic index (IPI) score.^5^, ^6^, ^7^, ^8^ Early ctDNA clearance during treatment has been associated with better response rates and superior survival outcomes in DLBCL patients.^5^, ^6^, ^7^, ^8^ However, further real‐world data as well as randomized clinical trials are needed to support full ctDNA clinical integration for personalized DLBCL therapy.^9^

Therefore, we have analysed 44 DLBCL patients (Table S1), all treated with R‐CHOP as a first‐line chemoimmunotherapy and determined the baseline ctDNA levels and its dynamics using the CAncer Personalized Profiling by deep Sequencing approach and a custom panel of 521 genes. Methodological details are described in Supporting Information S1.

The total plasma pretreatment cell‐free DNA (cfDNA) concentration strongly correlated with clinical stage, bulky disease, elevated LDH and IPI score (Figure S1). Without any threshold for input cfDNA quantity, the pretreatment baseline ctDNA was detectable in 32 patients (78% of 41 patients; Figure S2; Tables S2 and S3; analysis technically failed in three patients). Patients with detectable ctDNA had more advanced disease (Table S1) and higher plasma pretreatment levels of cfDNA (Figure S3A). Setting a threshold for minimal input cfDNA quantity (and associated quality) increased the proportion of ctDNA‐positive patients but excluded some with detectable ctDNA (Figure S3B). The median plasma ctDNA concentration at diagnosis was 1307 hGE/mL (human haploid genome equivalents per mL). Significantly higher levels of ctDNA were detected in patients with clinical stage III–IV, bulky disease ≥7.5 cm, PS ECOG (Eastern Cooperative Oncology Group performance status scale) 2–4, elevated LDH and IPI 3–5 (Figure 1; Table S4).

**FIGURE 1:**
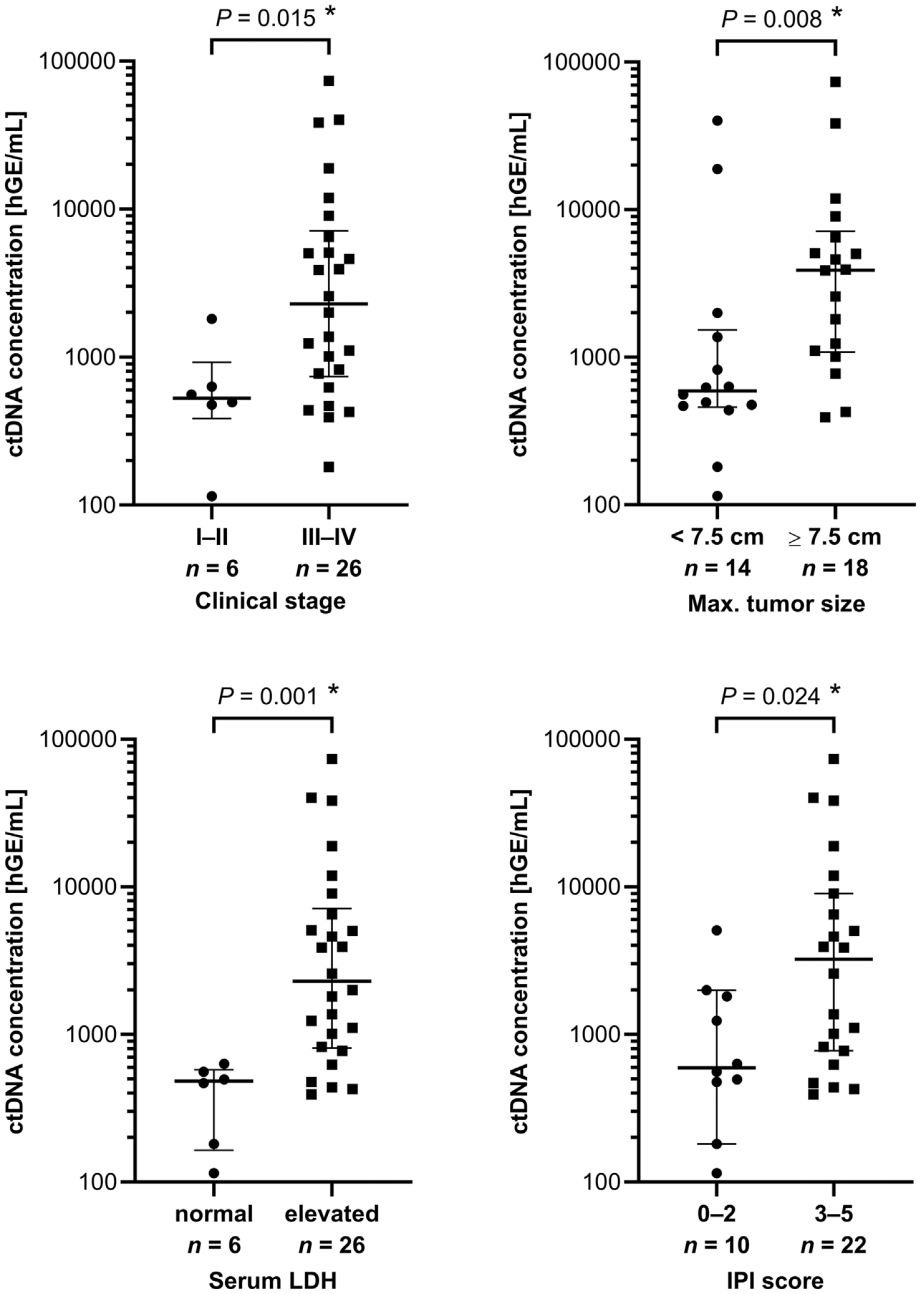
Higher pretreatment baseline ctDNA concentration is associated with advanced disease in diffuse large B‐cell lymphoma (DLBCL) patients. DLBCL patients were dichotomized based on clinical stage, maximal tumour size, serum lactate dehydrogenase and international prognostic index. Medians with interquartile ranges are displayed. Differences were assessed using Mann–Whitney *U*‐test. * marks statistically significant differences. ctDNA, circulating tumour DNA; hGE, human haploid genomic equivalent.

Based on the survival analysis, an optimal threshold of baseline ctDNA concentration was established at 5000 hGE/mL of plasma in accordance with the receiver operating curves to predict the end‐of‐treatment responses. Patients with lower baseline ctDNA levels had significantly better end‐of‐treatment overall response rate (ORR, 96% vs. 67%, *p* = 0.026; Table S5). Low baseline ctDNA patients had also significantly better progression free survival (PFS, 83% vs. 33% at 2 years, hazard ratio [HR] 0.27, 95% confidence interval [CI] 0.07–1.10, *p* = 0.012; Figure 2A) and a trend towards improved overall survival (OS, 87% vs. 78% at 2 years, HR 0.36, 95% CI 0.07–1.70, *p* = 0.125; Figure 2B). Multivariate analysis of dichotomized baseline ctDNA concentrations (<5000 vs. >5000 hGE/mL) and dichotomized IPI scores (3–5 vs. 0–2) indicated that ctDNA level at diagnosis is an independent risk factor for PFS (Table S6).

**FIGURE 2:**
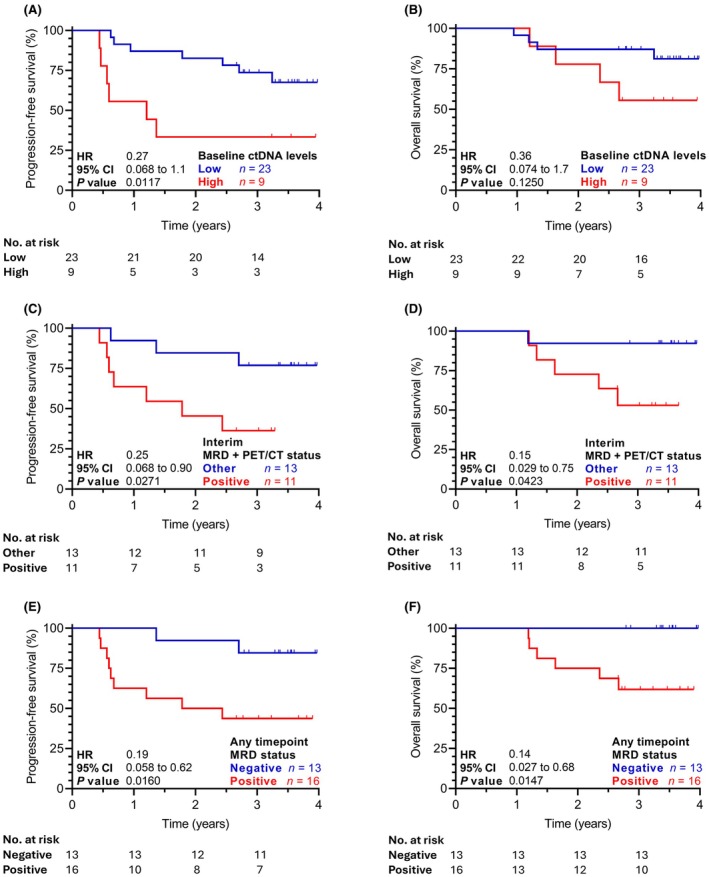
Lower baseline circulating tumour DNA (ctDNA) plasma concentration as well as minimal residual disease (MRD) negativity are associated with a better survival of diffuse large B‐cell lymphoma patients. (A) Comparison of progression‐free survival and (B) overall survival between patients with low and high pretreatment ctDNA concentrations (<5000 vs. >5000 hGE/mL respectively). (C) Progression‐free survival and (D) overall survival of patients that were MRD positive at interim restaging and, at the same time, did not reach complete remission at interim restaging by PET/CT. Survival of this group of patients is compared to all other patients. (E) Comparison of progression‐free survival and (F) overall survival between patients who reached MRD negativity at any time point (at interim and/or end of treatment restaging) versus MRD‐positive patients. Survival analyses were performed using the Kaplan–Meier estimator. A *p* value <0.05 was considered statistically significant. CI, confidence interval; HR, hazard ratio; MRD, minimal residual disease; PET/CT, positron emission tomography/computed tomography scan.

A ctDNA concentration decrease (baseline‐to‐interim) of more than 3 logs was associated with higher ORR at the end of the first‐line treatment (RR 100% vs. 67%, respectively, *p* = 0.042; Table S7). However, the baseline to interim 3‐log decrease of ctDNA concentration was not associated with improved survival rates (PFS *p* = 0.656, OS *p* = 0.437; Figure S4A,B). Additionally, a baseline to interim ctDNA concentration decrease of more than 2 logs was not associated with better treatment response rates (Table S7) or survival rates (PFS *p* = 0.844, OS *p* = 0.699; Figure S4C,D).

A baseline to end of treatment decrease of ctDNA concentration of more than 3 logs was associated with an improved complete remission rate (CR, 86% vs. 44%, *p* = 0.036; Table S8), though survival outcomes were not significantly different (PFS *p* = 0.443, OS *p* = 0.264; Figure S5A,B). A baseline‐to‐end of treatment ctDNA concentration decrease of at least 2 logs was associated with a better CR rate (82% vs. 33%, *p* = 0.025; Table S8) and better OS (94% vs. 50%, HR 0.18, 95% CI 0.02–1.5, *p* = 0.036), while the PFS was not different (PFS *p* = 0.346; Figure S5C,D).

Interim minimal residual disease (MRD) negativity was not associated with better treatment response rates as assessed by positron emission tomography/computed tomography scan (PET/CT) (Table S9), but it was associated with a trend towards improved PFS (89% vs. 53% at 2 years, HR 0.30, 95% CI 0.08–1.03, *p* = 0.101) and significantly better OS (100% vs. 67% at 2 years, HR 0.17, 95% CI 0.03–0.88, *p* = 0.034; Figure S6A,B). Importantly, 11 patients who remained MRD positive and at the same time did not reach CR according to the PET/CT scan (both at interim restaging) presented with a significantly inferior PFS (45% vs. 85% at 2 years, HR 4.00, 95% CI 1.10–15.0, *p* = 0.027; Figure 2C) as well as OS (73% vs. 91% at 2 years, HR 6.8, 95% CI 1.30–34.0, *p* = 0.043; Figure 2D).

End‐of‐treatment MRD negativity showed a trend towards association with a better ORR (100% vs. 69%, *p* = 0.105) and CR rate (90% vs. 54%, *p* = 0.062; Table S9), a trend towards association with improved PFS (2‐year PFS 90% vs. 46%, HR 0.26, 95% CI 0.07–0.95, *p* = 0.065), and was significantly associated with a better OS (2‐year OS 100% vs. 69%, HR 0.15, 95% CI 0.03–0.85, *p* = 0.031; Figure 6C,D).

Any time point MRD negativity was significantly associated with a prolonged PFS (92% vs. 50% at 2 years, HR 0.19, 95% CI 0.06–0.62, *p* = 0.016; Figure 2E) and OS (100% vs. 75% at 2 years, HR 0.14, 95% CI 0.03–0.68, *p* = 0.015; Figure 2F). Multivariate analysis of any time point MRD negativity together with end‐of‐treatment CR by imaging showed that the achievement of CR was an independent risk factor for PFS (HR 3.47, 95% CI 1.03–11.7, *p* = 0.045) and suggested any time point MRD negativity (HR 4.54, 95% CI 0.96–21.6, *p* = 0.057) as a potential independent risk factor.

As previously reported,^6^, ^8^, ^10^ we have found that higher pretreatment ctDNA levels at diagnosis are correlated with baseline DLBCL clinical characteristics related to a high tumour burden (including the well‐established IPI score) and are associated with inferior survival outcomes. Importantly, our analysis showed that high/low baseline ctDNA concentration is an independent prognostic factor for PFS when analysed alongside the IPI score. To stratify our patients into low‐ and high‐risk groups, we have used a threshold of 5000 hGE/mL of baseline plasma ctDNA. This threshold corresponds to approximately 3.7 log hGE/mL. In comparison, Kurtz et al. identified an optimal threshold of 2.5 log hGE/mL to stratify their cohort into low‐ and high‐risk patients.^5^ However, applying the 2.5 log hGE/mL cut‐off to our cohort classified only 6% of patients as low risk. Several studies failed to identify an association between baseline ctDNA levels and survival outcomes using the 2.5 log hGE/mL cut‐off^6^ or a threshold of 534 mean tumour molecules/mL.^7^ These discrepancies point to one of the most critical issues preventing fast implementation of ctDNA into routine clinical practice, the need for rigorous standardization from blood collection to bioinformatics data processing to define uniformly applicable thresholds.

Patients who achieved more than 3‐log decrease of ctDNA levels at interim restaging or at the end of the first‐line treatment had better treatment response rates, but their survival outcomes were not significantly different from those without such decrease. Moreover, the previously reported threshold of 2‐log decrease was even less predictive of survival outcomes in our cohort. Patients who achieved MRD negativity at any time point (interim or end of treatment) demonstrated significantly improved survival, suggesting that MRD negativity is a superior prognostic marker in comparison to ctDNA dynamics. Furthermore, multivariate analysis suggested that any time point MRD negativity could be an independent prognostic factor. This might be important in a situation where the end of treatment sample is not available.

Patients who were both MRD positive and PET/CT positive at interim restaging had significantly worse PFS and OS, consistently with previous reports.^5^, ^6^ Although the prognostic value of interim PET/CT alone is not yet fully clear, our findings suggest that combined interim assessments might correctly identify high‐risk patients who could potentially benefit from treatment intensification.

Limitations of our study are its size and a single centre/laboratory involvement, both affecting general applicability. The strengths include a real‐world design and a uniformly treated cohort with sufficient follow‐up. These strengths and limitations highlight the predictive power of ctDNA even within a small cohort of patients. Our data, thus, support findings that baseline ctDNA level and treatment‐related achievement of MRD negativity are valuable prognostic tools in DLBCL. Combined interim ctDNA and PET/CT assessment has a great potential to improve risk stratification in routine clinical practice.

## AUTHOR CONTRIBUTIONS

MT and OH were involved in the conceptualization of the study; JS, LD, MK, KP, JS, KB, MT and PK were responsible for sample collection and provided patients' data; PV, SM, JD, PB and TF oversaw clinical data collection; KL and SM were responsible for the samples' initial processing; PV, MT and OH performed clinical data correlations; IH performed optimization of ctDNA sequencing; IH, AV and KK performed sample preparation and sequencing; PZ and PN provided bioinformatics data processing; IH, AV, AT and OH did sequencing data evaluation; MT and OH provided funding; PV and OH wrote the manuscript; all authors reviewed, corrected and approved the manuscript.

## CONFLICT OF INTEREST STATEMENT

The authors report there are no competing interests to declare.

## ETHICS STATEMENT

Ethics approval was obtained from the local ethics committee and approved by the competent national authority. Written informed consent was obtained from each patient prior to the enrolment. The study was conducted in accordance with the rules of good clinical practice.

## Supporting information


Data S1.

## Data Availability

The datasets analysed in this study are available from the corresponding author upon reasonable request.
