# Supplementary material for: Circulating tumour DNA as a predictor of survival of patients with diffuse large B‐cell lymphoma in a daily practice
Source: Br J Haematol. 2025 Sep 7;207(5):2135–9. doi: 10.1111/bjh.70128 (PMC12624152; doi:10.1111/bjh.70128)
Supplement: Supplementary file 1 — Data S1. [file BJH-207-2135-s001.docx]

# SUPPLEMENTARY MATERIAL

**Title:** Circulating Tumor DNA as a Predictor of Survival of Patients with Diffuse Large B-cell Lymphoma in a Daily Practice

**Running Title:** Tracking ctDNA: A New Predictor of DLBCL Survival

**Authors:**

Prokop Vodicka^1,*^, Iva Hamova^1,2,*^, Adriana Velasova^2^, Kristyna Kupcova^1,2^, Petra Zemankova^3^, Petr Nehasil^3,4,5^, Anton Tkachenko^2^, Katerina Lochovska^1^, Sarka Muzikova^1^, Sarka Hrabetova^1,6^, Jitka Dlouha^1,6^, Petra Blahovcova^1,6^, Tomas Frouz^1^, Jana Senavova^1,2^, Lucie Dlouha^1^, Kamila Polgarova^1^, Magdalena Klanova^1,4^, Jana Salkova^1^, Katerina Benesova^1^, Pavel Klener^1,4^, Marek Trneny^1^, and Ondrej Havranek^1,2^

**Affiliations:**

^1^ First Department of Medicine-Hematology, First Faculty of Medicine, Charles University and General University Hospital, Prague, Czech Republic

^2^ BIOCEV, First Faculty of Medicine, Charles University, Vestec, Czech Republic

^3^ Institute of Medical Biochemistry and Laboratory Diagnostics, First Faculty of Medicine, Charles University and General University Hospital in Prague, Prague, Czech Republic

^4^ Institute of Pathological Physiology, First Faculty of Medicine, Charles University, Prague, Czech Republic

^5^ Department of Paediatrics and Inherited Metabolic Disorders, First Faculty of Medicine, Charles University and General University Hospital in Prague, Prague, Czech Republic

^6^ The Czech Lymphoma Study Group, Prague, Czech Republic

^*^ Equal contribution

**Correspondence to:**

Ondrej Havranek, BIOCEV, Prumyslova 595, 25250, Czech Republic, +420325873029, [ondrej.havranek@lf1.cuni.cz](mailto:ondrej.havranek@lf1.cuni.cz)

# SUPPLEMENTARY METHODS

*Study population and inclusion criteria*

Patients with previously untreated, systemic diffuse large B-cell lymphoma (DLBCL) diagnosed in a 1.5-year period between June 2020 and November 2021 and treated at a single academic center (First Department of Medicine – Department of Hematology, Charles University and General Hospital, Prague, Czech Republic) were included into the analysis. DLBCL diagnosis was confirmed according to the most recent World Health Organization classification of tumors.(1) Pathological evaluation of tumor samples was performed by Czech hematopathology reference centers and all findings were verified by expert hematopathologists. Individuals with central nervous system infiltration by DLBCL were excluded from this study. Additional inclusion criteria were: 1) availability of baseline ctDNA sample obtained before treatment initiation, 2) availability of buccal swab with sufficient germline DNA (gDNA) quantity, and 3) the R-CHOP therapy as a first-line treatment. Altogether, 44 DLBCL patients met these criteria and were eligible for analysis. In 9 of them no DNA alterations were identified in their cfDNA at baseline (ctDNA negative). Analysis failed in 3 patients for technical reasons. Written informed consent was obtained from each patient prior to the enrollment. The presented study adhered to the Declaration of Helsinki and was approved by the ethics committee of the General Hospital, Prague, Czech Republic.

*Clinical characteristics and determination of response rates by imaging*

Clinical data were prospectively collected within the Czech non-Hodgkin lymphoma registry “NiHiL” (NCT03199066) and verified in medical records. These included age, gender, clinical stage (based on the Ann Arbor staging system), number of extranodal sites, presence of bone marrow involvement, presence of bulky disease (≥ 7.5 cm), performance status (according to the Eastern Cooperative Oncology Group, PS ECOG)(2), serum levels of LDH (lactate dehydrogenase), IPI (international prognostic index)(3), DLBCL subtype according to the Hans’ algorithm (germinal center B-cell like/GCB vs. non-GCB)(4), and a time from diagnosis to the treatment initiation. Treatment response rates were assessed by PET/CT (or CT alone) at interim restaging and at the end of the first-line treatment. The following categories were consequently used for further evaluation: overall response rate (ORR), complete remission (CR), partial remission (PR), stable disease (SD), and progressive disease (PD).(5)

We have included 44 DLBCL patients, all treated with R-CHOP as a first line chemoimmunotherapy (Supplementary Table 1). Within 32 patients with detectable ctDNA (median age 65.5 years, range 40–80 years), 22 (69%) were male, clinical stage III–IV disease was detected in 26 (81%) cases, more than one extranodal site was involved in 19 (59%) cases, and bone marrow in 11 (34%) patients. Bulky disease of ≥ 7.5 cm was present in 18 (56%) cases, performance status according to Eastern Cooperative Oncology Group (PS ECOG) 2–4 in 6 (19%) patients, elevated serum LDH levels in 26 (81%) patients, IPI of 3–5 in 22 (69%) patients, and 17 (53%) cases had non-germinal center B-cell like DLBCL subtype. The median time from diagnosis to treatment initiation was 27 days (range 7–73 days, Supplementary Table 2). The median number of R-CHOP treatment cycles was 6 (range 4–8), with 26 (81%) patients completing the intended treatment (≥ 6 cycles of R-CHOP). At interim and end-of-treatment restaging, the overall response rate (ORR) was 84% and 88%, respectively, and the CR rate was 25% and 72%, respectively (Supplementary Table 3). The median follow-up of the 32 patients with detectable ctDNA was 3.5 years; 2-year PFS was 69%, and 2-year OS was 84%. There were no statistically significant differences between the group of all DLBCL patients (*n* = 44) and patients with detected baseline ctDNA (*n* = 32).

*Samples collection and DNA extraction*

Blood samples were collected prior to the treatment initiation, at interim restaging, and after first-line therapy completion. Peripheral blood samples were collected using Cell-Free DNA BCT^®^ tubes (Streck) and plasma was separated according to the manufacturer’s protocol (two centrifugation steps at 1,600 g for 10 minutes). Supernatants were consequently frozen at –80 °C for further processing. Buccal swabs (using iSWAB DNA Collection Kit, 1.0ml, Mawi) were collected at diagnosis as a source of control background gDNA.

Extraction of cfDNA was done by QIAamp MinElute ccfDNA Midi Kit (Quiagen). gDNA was extracted using Wizard® Genomic DNA Purification Kit (Promega). Quantity and quality of extracted DNA was measured using Quant-iTTM PicoGreen dsDNA Assay kit (Thermo Fisher Scientific), Qubit™ dsDNA HS Assay Kit (CarolinaBioSystems), and Bioanalyzer 2100 High Sensitivity DNA Kit (Agilent). All kits were used as per the manufacturers’ protocol.

*ctDNA levels assessment*

Plasma ctDNA levels were assessed using a CAPP-Seq (CAncer Personalized Profiling by deep Sequencing) approach using a custom panel targeting coding regions of 521 genes frequently altered in lymphomas. The sequencing library preparation steps started with mechanic fragmentation of gDNA (microTUBE-50 AFA Fiber Screw-Cap, Covaris) to match naturally fragmented cfDNA. Next, 100 ng of fragmented gDNA and 50 ng of ctDNA were used to perform samples indexing and amplification using KAPA HyperPlus Kit (Roche) with KAPA Universal UMI adapters (Roche) and KAPA UDI Primer Mixes (Roche). Following DNA quantification (Qubit™ dsDNA HS Assay Kit, CarolinaBioSystems) and quality assessment (Bioanalyzer, Agilent), indexed samples were mixed in equimolar ratios to perform target enrichment using KAPA HyperCapture Reagent Kit (Roche), all according to the manufacturers’ protocols. Resulting pooled libraries were sequenced using NovaSeq platform (Illumina) for the target coverage at least 2,000 reads at 80 % of targeted regions.

Following standard NGS data pre-processing and reads alignment (including PCR duplication removal based on used unique molecular identifiers – UMI), DNA variants were called using VarScan 2(6) in paired gDNA and ctDNA samples with the following parameters: --min-var-freq 0.001, --min-coverage-normal 5, --min-coverage-tumor 1, --min-freq-for-hom 0.75, --normal-purity 1, --tumor-purity 0.3, --somatic-p-value 0.0001, --strand-filter 1.

Average variant allele frequency of identified DNA alterations was used to estimate the proportion of ctDNA within cfDNA. Resulting ctDNA concentration (in ng/mL of plasma) was converted to the human haploid genome equivalents (hGE) per mL of plasma as previously described.(7) Serial ctDNA measurements were compared on a 10-logarithmic (log) scale and minimal residual disease (MRD) negativity was defined as no detectable patient specific DNA alterations in interim or end of treatment cfDNA sample.

*Statistical analysis*

Categorical variables were presented as counts and percentages, while continuous variables were described using medians, ranges, or interquartile ranges (IQR). Differences between categorical variables were assessed by Pearson’s χ^2^ or Fisher’s Exact Test. Mann-Whitney U test was used for comparison between continuous variables. Survival analyses were performed using the Kaplan-Meier estimator and the Cox regression and described with a hazard ratio (HR) and a 95% confidence interval (95% CI). Progression-free survival (PFS) was defined as the time from diagnosis to relapse, progression, death, or last follow-up. The overall survival (OS) was defined as the time from diagnosis to death or last follow-up. A *P* value < 0.05 was considered statistically significant. Analyses were performed using R Statistical Software (v4.3.3; R Core Team 2024) and GraphPad Prism (version 8 for Windows, GraphPad Software, Boston, Massachusetts, USA).

# SUPPLEMENTARY TABLES

|  |  | **All patients** | | **Patients without detected ctDNA** | | **Patients with detected ctDNA** | | ***P* value** |
| --- | --- | --- | --- | --- | --- | --- | --- | --- |
|  |  | ***n*** | ***%*** | ***n*** | ***%*** | ***n*** | ***%*** |  |
| **Number of patients** |  | 44 |  | 12 |  | 32 |  |  |
| **Age** | median years, range | 62 | 51–71 | 53 | 33–72 | 65.5 | 40–80 | 0.020 * |
| **Age category** | ≤ 60 years | 20 | 45.5 | 9 | 75.0 | 11 | 34.4 | 0.116 |
|  | > 60 years | 24 | 54.5 | 3 | 25.0 | 21 | 65.6 |  |
| **Gender** | male | 25 | 56.8 | 3 | 25.0 | 22 | 68.8 | 0.009 * |
|  | female | 19 | 43.2 | 9 | 75.0 | 10 | 31.3 |  |
| **Clinical stage** | I or II | 13 | 29.5 | 7 | 58.3 | 6 | 18.8 | 0.010 * |
|  | III or IV | 31 | 70.5 | 5 | 41.7 | 26 | 81.3 |  |
| **No. of extranodal sites** | 0 | 7 | 15.9 | 3 | 25.0 | 4 | 12.5 | 0.805 |
|  | 1 | 13 | 29.5 | 4 | 33.3 | 9 | 28.1 |  |
|  | ≥ 2 | 24 | 54.5 | 5 | 41.7 | 19 | 59.4 |  |
| **BM involvement** | no | 33 | 75.0 | 12 | 100.0 | 21 | 65.6 | 0.021 * |
|  | yes | 11 | 25.0 | 0 | 0.0 | 11 | 34.4 |  |
| **Bulky disease ≥ 7.5 cm** | no | 22 | 50.0 | 8 | 66.7 | 14 | 43.8 | 0.176 |
|  | yes | 22 | 50.0 | 4 | 33.3 | 18 | 56.3 |  |
| **PS ECOG** | 0 or 1 | 34 | 77.3 | 8 | 66.7 | 26 | 81.3 | 0.554 |
|  | 2 | 8 | 18.2 | 3 | 25.0 | 5 | 15.6 |  |
|  | 3 or 4 | 2 | 4.5 | 1 | 8.3 | 1 | 3.1 |  |
| **LDH level** | not elevated | 13 | 29.5 | 7 | 58.3 | 6 | 18.8 | 0.010 * |
|  | elevated | 31 | 70.5 | 5 | 41.7 | 26 | 81.3 |  |
| **IPI score** | low | 12 | 27.3 | 7 | 58.3 | 5 | 15.6 | 0.012 * |
|  | low-intermed. | 7 | 15.9 | 2 | 16.7 | 5 | 15.6 |  |
|  | high-intermed. | 6 | 13.6 | 0 | 0.0 | 6 | 18.8 |  |
|  | high | 19 | 43.2 | 3 | 25.0 | 16 | 50.0 |  |
| **Cell of origin** | GCB | 23 | 52.3 | 8 | 66.7 | 15 | 46.9 | 0.242 |
|  | non-GCB | 21 | 47.7 | 4 | 33.3 | 17 | 53.1 |  |
| **Diagnosis to treatment interval** | median days, range | 30 | 22–41 | 31.5 | 1–90 | 27 | 1–73 | 0.171 |

## **Supplementary Table 1.**

**Clinical characteristics of all DLBCL patients and in subgroups based on ctDNA detection status.** The left part of the table shows all DLBCL patients. The right part of the table shows a comparison of clinical characteristics between patients with and without detected ctDNA Differences were evaluated using the Pearson’s χ2 test or the Fisher’s Exact Test. * marks statistically significant difference. BM, bone marrow; ctDNA, circulating tumor DNA; GCB, germinal center B-cell like; hGE, human haploid genomic equivalent; IPI, International Prognostic Index, LDH, lactate dehydrogenase; PS ECOG, performance status according to the Eastern Cooperative Oncology Group.

|  |  | **Patients with detected ctDNA** | | **Patients as divided based on the baseline ctDNA levels** | | | | ***P* value** |
| --- | --- | --- | --- | --- | --- | --- | --- | --- |
|  |  |  |  | **< 5,000 hGE/mL** | | **> 5,000 hGE/mL** | |  |
|  |  | ***n*** | **%** | ***n*** | **%** | ***n*** | **%** |  |
| **Number of patients** |  | 32 |  | 23 |  | 9 |  |  |
| **Age** | median (range) | 65.5 | 40–80 | 64 | 40–80 | 68 | 48–79 | 0.239 |
| **Age category** | ≤ 60 years | 11 | 34.4 | 9 | 39.1 | 2 | 22.2 | 0.365 |
|  | > 60 years | 21 | 65.6 | 14 | 60.9 | 7 | 77.8 |  |
| **Gender** | male | 22 | 68.8 | 15 | 65.2 | 7 | 77.8 | 0.491 |
|  | female | 10 | 31.3 | 8 | 34.8 | 2 | 22.2 |  |
| **Clinical stage** | I or II | 6 | 18.8 | 6 | 26.1 | 0 | 0.0 | 0.149 |
|  | III or IV | 26 | 81.3 | 17 | 73.9 | 9 | 100 |  |
| **No. of extranodal sites** | 0 | 4 | 12.5 | 4 | 17.4 | 0 | 0.0 | 0.304 |
|  | 1 | 9 | 28.1 | 5 | 21.7 | 4 | 44.4 |  |
|  | ≥ 2 | 19 | 59.4 | 14 | 60.9 | 5 | 55.6 |  |
| **BM involvement** | no | 21 | 65.6 | 17 | 73.9 | 4 | 44.1 | 0.213 |
|  | yes | 11 | 34.4 | 6 | 26.1 | 5 | 55.6 |  |
| **Bulky disease ≥ 7.5 cm** | no | 14 | 43.8 | 12 | 52.2 | 2 | 22.2 | 0.235 |
|  | yes | 18 | 56.3 | 11 | 47.8 | 7 | 77.8 |  |
| **PS ECOG** | 0 or 1 | 26 | 81.3 | 23 | 100 | 3 | 33.3 | 0.281 |
|  | 2 | 5 | 15.6 | 0 | 0.0 | 5 | 55.6 |  |
|  | 3 or 4 | 1 | 3.1 | 0 | 0.0 | 1 | 11.1 |  |
| **LDH level** | not elevated | 6 | 18.8 | 6 | 26.1 | 0 | 0.0 | 0.149 |
|  | elevated | 26 | 81.3 | 17 | 73.9 | 9 | 100 |  |
| **IPI score** | low | 5 | 15.6 | 5 | 21.7 | 0 | 0.0 | 0.211 |
|  | low-intermed. | 5 | 15.6 | 4 | 17.4 | 1 | 11.1 |  |
|  | high-intermed. | 6 | 18.8 | 4 | 17.4 | 2 | 22.2 |  |
|  | high | 16 | 50.0 | 10 | 43.5 | 6 | 66.7 |  |
| **Cell of origin** | GCB | 15 | 46.9 | 11 | 47.8 | 4 | 44.4 | 0.999 |
|  | non-GCB | 17 | 53.1 | 12 | 52.2 | 5 | 55.6 |  |
| **Diagnosis to treatment interval** | median days (range) | 27 | 7–73 | 29 | 12–73 | 25 | 7–42 | 0.046 * |

## Supplementary Table 2.

**Clinical characteristics of DLBCL patients with detected ctDNA.** The left part of the table shows summary information. The right part of the table shows a comparison of clinical characteristics between patients with low and high ctDNA levels at the time of diagnosis (< 5,000 hGE/mL versus > 5,000 hGE/mL) with results of statistical significance evaluation. Differences were evaluated using the Pearson’s χ^2^ test or the Fisher’s Exact Test. * marks statistically significant difference. BM, bone marrow; ctDNA, circulating tumor DNA; GCB, germinal center B-cell like; hGE, human haploid genomic equivalent; IPI, International Prognostic Index, LDH, lactate dehydrogenase; PS ECOG, performance status according to the Eastern Cooperative Oncology Group.

|  | **Interim** | | **End-of-treatment** | |
| --- | --- | --- | --- | --- |
|  | *n* | % | *n* | % |
| **Overall response** | 27 | 84.4 | 28 | 87.5 |
| Complete remission | 8 | 25.0 | 23 | 71.9 |
| Partial remission | 19 | 59.4 | 5 | 15.6 |
| **Stable disease** | 1 | 3.1 | 0 | 0 |
| **Progressive disease** | 0 | 0 | 4 | 12.5 |
| **Not evaluated** | 4 | 12.5 | 0 | 0 |

## Supplementary Table 3.

**Treatment response rates of DLBCL patients with detected baseline ctDNA** (*n* = 32). Treatment response rates were evaluated at interim and end-of-treatment restaging by a standard positron emission tomography/computed tomography (PET/CT) imaging.

|  | **Group 1** | | **Group 2** | | ***P* value** |
| --- | --- | --- | --- | --- | --- |
|  | *n* | *median ctDNA level [hGE/mL]* | *n* | *median ctDNA level [hGE/mL]* |  |
| **Clinical stage** I–II vs. III–IV | 6 | 528 | 26 | 2,291 | 0.015 * |
| **Extranodal sites** 0–1 vs. ≥2 | 13 | 1,240 | 19 | 1,373 | 0.439 |
| **Bone marrow involvement** no vs. yes | 21 | 1,111 | 11 | 3,927 | 0.217 |
| **Bulky** no vs. yes | 14 | 592 | 18 | 3,899 | 0.008 * |
| **Performance status ECOG** 0–1 vs. 2–4 | 26 | 918 | 6 | 10,481 | 0.001 * |
| **Lactate dehydrogenase** normal vs. elevated | 6 | 482 | 26 | 2,291 | 0.001 * |
| **International Prognostic Index** 0–2 vs. 3–5 | 10 | 596 | 22 | 3,228 | 0.024 * |
| **Cell of origin** GCB vs. non-GCB | 15 | 1,013 | 17 | 1,813 | 0.355 |
| **Diagnosis-to-treatment interval** < 28 vs. ≥ 28 days | 17 | 3,871 | 15 | 625 | 0.057 |

## Supplementary Table 4.

**Comparison of pre-treatment baseline ctDNA levels in DLBCL patients as divided to two groups according to selected clinical characteristics.** DLBCL patients were dichotomized based on their clinical characteristics as outlined in the table and differences in ctDNA levels were compared by the Mann-Whitney U test. * marks statistically significant differences. ECOG, Eastern Cooperative Oncology Group; GCB, germinal center B-cell like; hGE, human haploid genomic equivalent.

|  |  | **ctDNA level at diagnosis** | | | | |
| --- | --- | --- | --- | --- | --- | --- |
|  |  | **< 5,000 hGE/mL** | | **> 5,000 hGE/mL** | | ***P* value** |
|  |  | n | % | n | % |  |
| **End-of-treatment response rates** | **Number of patients** | 23 |  | 9 |  |  |
|  | **Overall response rate** | 22 | 95.7 | 6 | 66.7 | 0.026 *  0.682 |
|  | Complete remission | 17 | 73.9 | 6 | 66.7 |  |
|  | Partial remission | 5 | 21.7 | 0 | 0.0 |  |
|  | **Stable disease** | 0 | 0.0 | 0 | 0.0 |  |
|  | **Progressive disease** | 1 | 4.3 | 3 | 33.3 |  |

## Supplementary Table 5.

**Plasma ctDNA concentration at diagnosis predicts treatment outcome.** Treatment response rates at the end of the first-line treatment are compared between patients with low and high ctDNA concentration the time of diagnosis (< 5,000 hGE/mL versus > 5,000 hGE/mL). Differences in response rates were assessed by Pearson’s χ^2^ Test. * marks statistically significant difference. ctDNA, circulating tumor DNA; hGE, human haploid genomic equivalent.

|  | | ***P* value** | **HR** | **95% CI** |
| --- | --- | --- | --- | --- |
| **PFS** | **ctDNA** high vs. low | 0.036 * | 3.30 | 1.08– 10.1 |
|  | **IPI** 3–5 vs. 0–2 | 0.201 | 2.72 | 0.59–12.5 |
| **OS** | **ctDNA** high vs. low | 0.167 | 2.78 | 0.65–11.8 |
|  | **IPI** 3–5 vs. 0–2 | 0.902 | 1.11 | 0.21–5.87 |

## Supplementary Table 6.

**ctDNA level at diagnosis is an independent risk factor for progression free survival (PFS).** Results of multivariate analysis are displayed for patients dichotomized according to the International Prognostic Index (3–5 versus 0–2) together with patients dichotomized according to the ctDNA levels at the time of diagnosis (high > 5,000 hGE/mL versus low < 5,000 hGE/mL). Analysis was performed using the Cox regression model and described with a hazard ratio (HR) and a 95% confidence interval (95% CI). * marks statistically significant difference. ctDNA, circulating tumor DNA; hGE, human haploid genomic equivalent; IPI, International Prognostic Index; OS, overall survival; PFS, progression-free survival.

|  |  | **Baseline-to-interim**  **3-log ctDNA decrease** | | | | | **Baseline-to-interim**  **2-log ctDNA decrease** | | | | |
| --- | --- | --- | --- | --- | --- | --- | --- | --- | --- | --- | --- |
|  |  | **yes** | | **no** | | ***P* value** | **yes** | | **no** | | ***P* value** |
|  |  | *n* | % | *n* | % |  | *n* | % | *n* | % |  |
|  | **Number of patients** | 15 |  | 9 |  |  | 17 |  | 7 |  |  |
| **Interim PET/CT**  **response rates** | **Overall response** | 14 | 93.3 | 7 | 77.8 | 0.533 | 16 | 94.1 | 5 | 71.4 | 0.127 |
|  | Complete remission | 4 | 26.7 | 3 | 33.3 | 0.728 | 5 | 29.4 | 2 | 28.6 | 0.967 |
|  | Partial remission | 10 | 66.7 | 4 | 44.4 |  | 11 | 64.7 | 3 | 42.9 |  |
|  | **Stable disease** | 0 | 0 | 1 | 11.1 |  | 0 | 0 | 1 | 14.3 |  |
|  | **Progressive disease** | 0 | 0 | 0 | 0 |  | 0 | 0 | 0 | 0 |  |
|  | **Not evaluated** | 1 | 6.7 | 1 | 11.1 |  | 1 | 5.9 | 1 | 14.3 |  |
| **End-of-treatment PET/CT response rates** | **Overall response** | 15 | 100 | 6 | 66.7 | 0.042 * | 16 | 94 | 5 | 71.4 | 0.127 |
|  | Complete remission | 13 | 86.7 | 4 | 44.4 | 0.028 * | 14 | 82.4 | 3 | 42.9 | 0.053 |
|  | Partial remission | 2 | 13.3 | 2 | 22.2 |  | 2 | 11.8 | 2 | 28.6 |  |
|  | **Stable disease** | 0 | 0 | 0 | 0 |  | 0 | 0 | 0 | 0 |  |
|  | **Progressive disease** | 0 | 0 | 3 | 33.3 |  | 1 | 6 | 2 | 28.6 |  |
|  | **Not evaluated** | 0 | 0 | 0 | 0 |  | 0 | 0 | 0 | 0 |  |

## Supplementary Table 7.

**Greater baseline**-**to-interim decrease of ctDNA concentration is associated with a better treatment response rate.** Treatment response rates (at interim and at the end of treatment, evaluated by positron emission tomography/computed tomography imaging, i.e., PET/CT) were compared between patients with greater than 3-log and lower than 3-log decrease of baseline-to-interim ctDNA concentrations and between patients with greater than 2-log and lower than 2-log decrease of baseline-to-interim ctDNA concentrations. Differences were evaluated using the Pearson’s χ^2^ test or the Fisher’s Exact Test. * marks statistically significant differences. ctDNA, circulating tumor DNA.

|  |  | **Baseline-to-end of treatment**  **3-log ctDNA decrease** | | | | | **Baseline-to-end of treatment**  **2-log ctDNA decrease** | | | | |
| --- | --- | --- | --- | --- | --- | --- | --- | --- | --- | --- | --- |
|  |  | **yes** | | **no** | | ***P* value** | **yes** | | **no** | | ***P* value** |
|  |  | *n* | % | *n* | % |  | *n* | % | *n* | % |  |
|  | **Number of patients** | 14 |  | 9 |  |  | 17 |  | 6 |  |  |
| **Interim PET/CT**  **response rates** | **Overall response** | 12 | 85.7 | 7 | 77.8 | 0.624 | 15 | 88.2 | 4 | 66.7 | 0.231 |
|  | Complete remission | 5 | 35.7 | 3 | 33.3 | 0.907 | 6 | 35.3 | 2 | 33.3 | 0.931 |
|  | Partial remission | 7 | 50.0 | 4 | 44.4 |  | 9 | 52.9 | 2 | 33.3 |  |
|  | **Stable disease** | 0 | 0 | 1 | 11.1 |  | 0 | 0 | 1 | 16.7 |  |
|  | **Progressive disease** | 0 | 0 | 0 | 0 |  | 0 | 0 | 0 | 0 |  |
|  | **Not evaluated** | 2 | 14.3 | 1 | 11.1 |  | 2 | 11.8 | 1 | 16.7 |  |
| **End-of-treatment PET/CT response rates** | **Overall response** | 13 | 92.9 | 6 | 66.7 | 0.106 | 15 | 88.2 | 4 | 66.7 | 0.231 |
|  | Complete remission | 12 | 85.7 | 4 | 44.4 | 0.036 * | 14 | 82.4 | 2 | 33.3 | 0.025 * |
|  | Partial remission | 1 | 7.1 | 2 | 22.2 |  | 1 | 5.9 | 2 | 33.3 |  |
|  | **Stable disease** | 0 | 0 | 0 | 0 |  | 0 | 0 | 0 | 0 |  |
|  | **Progressive disease** | 1 | 7.1 | 3 | 33.3 |  | 2 | 11.8 | 2 | 33.3 |  |
|  | **Not evaluated** | 0 | 0 | 0 | 0 |  | 0 | 0 | 0 | 0 |  |

## Supplementary Table 8.

**Baseline**-**to-end of treatment decrease of ctDNA concentration is associated with better treatment response rates.** Treatment response rates (at interim and at the end of treatment, evaluated by positron emission tomography/computed tomography imaging, i.e., PET/CT) were compared between patients with greater than 3-log and lower than 3-log decrease of baseline-to-end of treatment ctDNA concentrations and between patients with greater than 2-log and lower than 2-log decrease of baseline-to-end of treatment ctDNA concentrations. Differences were evaluated using the Pearson’s χ^2^ test or the Fisher’s Exact Test. * marks statistically significant differences. ctDNA, circulating tumor DNA.

|  |  | **Interim**  **MRD negativity** | | | | | **End-of-treatment**  **MRD negativity** | | | | | **Any timepoint**  **MRD negativity** | | | | |
| --- | --- | --- | --- | --- | --- | --- | --- | --- | --- | --- | --- | --- | --- | --- | --- | --- |
|  |  | **yes** | | **no** | | ***P* value** | **yes** | | **no** | | ***P* value** | **yes** | | **no** | | ***P* value** |
|  |  | *n* | % | *n* | % |  | *n* | % | *n* | % |  | *n* | % | *n* | % |  |
|  | **Number of patients** | 9 |  | 15 |  |  | 10 |  | 13 |  |  | 13 |  | 16 |  |  |
| **Interim PET/CT**  **response rates** | **Overall response** | 9 | 100 | 12 | 80.0 | 0.266 | 8 | 80.0 | 11 | 84.6 | 0.999 | 11 | 84.6 | 14 | 87.5 | 0.823 |
|  | Complete remission | 4 | 44.4 | 3 | 20.0 | 0.202 | 4 | 40.0 | 4 | 30.8 | 0.645 | 5 | 38.5 | 3 | 18.8 | 0.238 |
|  | Partial remission | 5 | 55.6 | 9 | 60.0 |  | 4 | 40.0 | 7 | 53.8 |  | 6 | 46.2 | 11 | 68.8 |  |
|  | **Stable disease** | 0 | 0 | 1 | 6.7 |  | 0 | 0 | 1 | 7.7 |  | 0 | 0 | 1 | 6.3 |  |
|  | **Progressive disease** | 0 | 0 | 0 | 0 |  | 0 | 0 | 0 | 0 |  | 0 | 0 | 0 | 0 |  |
|  | **Not evaluated** | 0 | 0 | 2 | 13.3 |  | 2 | 20.0 | 1 | 7.7 |  | 2 | 15.4 | 1 | 6.3 |  |
| **End-of-treatment PET/CT response rates** | **Overall response** | 9 | 100 | 12 | 80.0 | 0.266 | 10 | 100 | 9 | 69.2 | 0.105 | 13 | 100 | 12 | 75.0 | 0.106 |
|  | Complete remission | 8 | 88.9 | 9 | 60.0 | 0.132 | 9 | 90.0 | 7 | 53.8 | 0.062 | 11 | 84.6 | 9 | 56.3 | 0.129 |
|  | Partial remission | 1 | 11.1 | 3 | 20.0 |  | 1 | 10.0 | 2 | 15.4 |  | 2 | 15.4 | 3 | 18.8 |  |
|  | **Stable disease** | 0 | 0.0 | 0 | 0 |  | 0 | 0 | 0 | 0 |  | 0 | 0 | 0 | 0 |  |
|  | **Progressive disease** | 0 | 0.0 | 3 | 20.0 |  | 0 | 0 | 4 | 30.8 |  | 0 | 0 | 4 | 25.0 |  |
|  | **Not evaluated** | 0 | 0 | 0 | 0 |  | 0 | 0 | 0 | 0 |  | 0 | 0 | 0 | 0 |  |

## Supplementary Table 9.

**Minimal residual disease (MRD) ctDNA status is not associated with treatment response rates.** Treatment response rates (at interim and at the end of treatment, evaluated by positron emission tomography/computed tomography imaging, i.e., PET/CT) were compared between patients who achieved MRD negativity at interim restaging, at the end of treatment, or any timepoint during therapy. Differences were evaluated using the Pearson’s χ^2^ test or the Fisher’s Exact Test. * marks statistically significant differences. ctDNA, circulating tumor DNA.

# SUPPLEMENTARY FIGURES

## Supplementary Figure 1.

**Pretreatment cell free DNA (cfDNA) concentration is associated with advanced disease in DLBCL patients.** DLBCL patients were dichotomized based on clinical stage, maximal tumor size, serum lactate dehydrogenase (LDH) and international prognostic index (IPI). Medians with interquartile ranges are displayed. Differences were assessed using Mann-Whitney U test. P < 0.05 - statistically significant differences. cfDNA, cell free DNA.


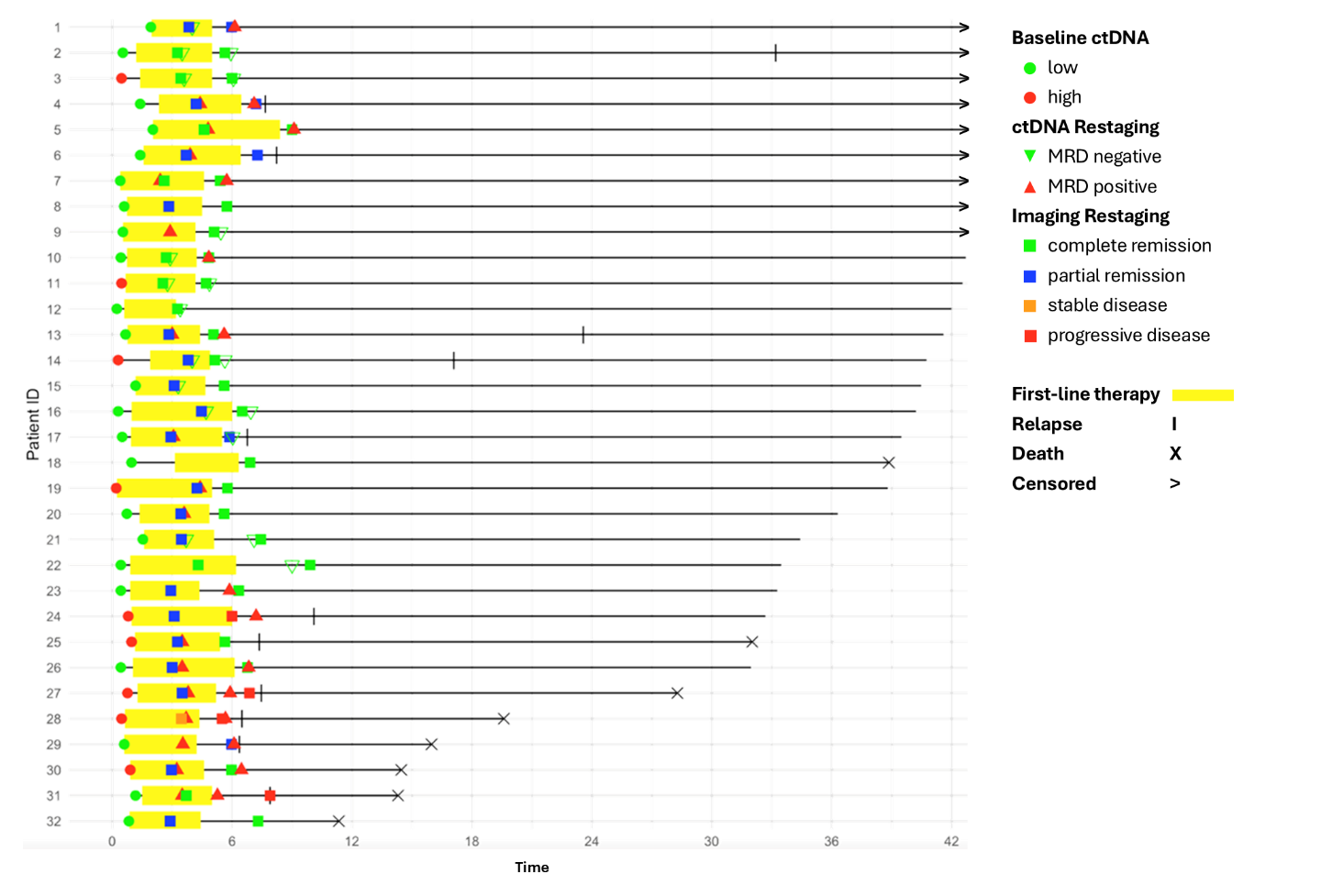


## Supplementary Figure 2.

**Swimmers’ plot of all patients with good quality cfDNA samples and pre-treatment detectable ctDNA who were included in the analysis** (*n* = 32). Categories of ctDNA pre-treatment concentrations (high > 5,000 hGE/mL versus low < 5,000 hGE/mL), MRD status, as well as PET/CT-based restaging results and events are displayed. ctDNA, circulating tumor DNA; hGE, human haploid genomic equivalent; MRD, minimal residual disease.


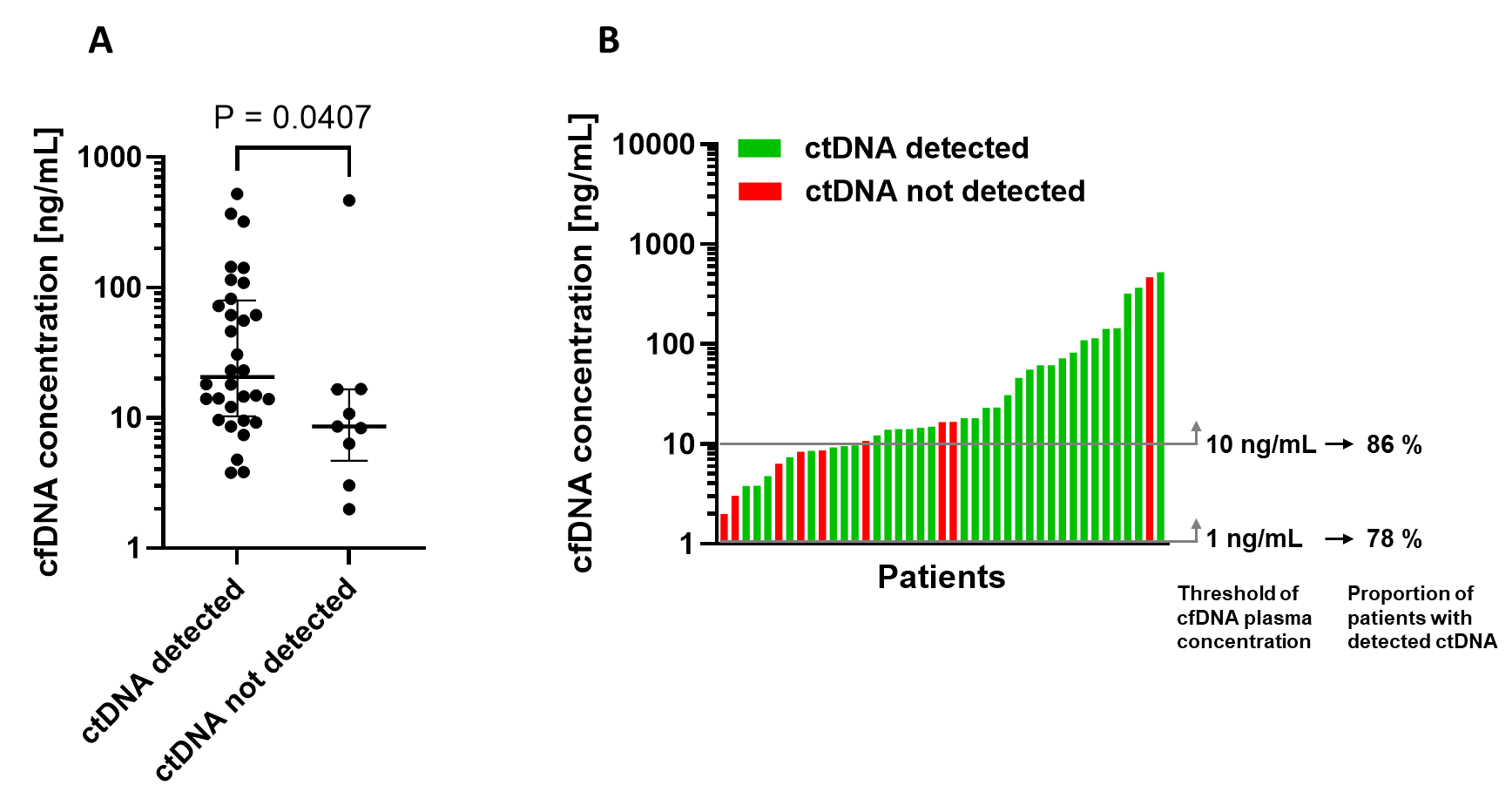


## Supplementary Figure 3.

**Circulating tumor DNA detectability is determined by plasma cell free DNA concentration.** **(A)** Patients with pre-treatment detectable circulating tumor DNA (ctDNA) had higher concentrations of pre-treatment plasma total cell free DNA (cfDNA). Median and interquartile range are displayed. **(B)** Pre-treatment plasma cfDNA concentration threshold determines ctDNA detectability. With no threshold, ctDNA was detected in 78% of patients. Raising the input threshold to 10 ng/mL raises the detectability of ctDNA to 86%. In both panels, results of 41 patients are displayed, excluding three patients with technically failed analysis. Differences were assessed using Mann-Whitney U test. P < 0.05 - statistically significant differences. cfDNA, cell free DNA; ctDNA, circulating tumor DNA.

## Supplementary Figure 4.

**Greater baseline-to-interim decrease of ctDNA concentration is not associated with better survival.** (**A**) Progression free survival and (**B**) overall survival of patients with higher than 3-log baseline-to-interim ctDNA concentration decrease in comparison to patients with lower baseline-to-interim ctDNA decrease. (**C**) Progression free survival and (**D**) overall survival of patients with higher than 2-log baseline-to-interim ctDNA concentration decrease in comparison to patients with lower baseline-to-interim ctDNA decrease. Survival analyses were performed using the Kaplan-Meier estimator. A *P* value < 0.05 was considered statistically significant. CI, confidence interval; ctDNA, circulating tumor DNA; HR, hazard ratio.

## Supplementary Figure 5.

**Baseline-to-end of treatment decrease of ctDNA concentration might be associated with a better survival.** (**A**) Progression free survival and (**B**) overall survival of patients with higher than 3-log baseline-to-end of treatment ctDNA concentration decrease in comparison to patients with lower baseline-to-end of treatment ctDNA decrease. (**C**) Progression free survival and (**D**) overall survival of patients with higher than 2-log baseline-to-end of treatment ctDNA concentration decrease in comparison to patients with lower baseline-to-end of treatment ctDNA decrease. Survival analyses were performed using the Kaplan-Meier estimator. A *P* value < 0.05 was considered statistically significant. CI, confidence interval; ctDNA, circulating tumor DNA; HR, hazard ratio.

## Supplementary Figure 6.

**Achievement of minimal residual disease (MRD) negativity is associated with a better survival.** (**A**) Progression free survival and (**B**) overall survival of patients that were MRD negative versus MRD positive at interim restaging. (**C**) Progression free survival and (**D**) overall survival of patients that were MRD negative versus MRD positive at end-of-treatment restaging. Survival analyses were performed using the Kaplan-Meier estimator. A *P* value < 0.05 was considered statistically significant. CI, confidence interval; HR, hazard ratio.

# SUPPLEMENTARY REFERENCES

1. Swerdlow SH, Campo E, Pileri SA, Harris NL, Stein H, Siebert R, et al. The 2016 revision of the World Health Organization classification of lymphoid neoplasms. Blood. 2016;127(20):2375-90.

2. Oken MM, Creech RH, Tormey DC, Horton J, Davis TE, McFadden ET, et al. Toxicity and response criteria of the Eastern Cooperative Oncology Group. Am J Clin Oncol. 1982;5(6):649-55.

3. A predictive model for aggressive non-Hodgkin's lymphoma. N Engl J Med. 1993;329(14):987-94.

4. Hans CP, Weisenburger DD, Greiner TC, Gascoyne RD, Delabie J, Ott G, et al. Confirmation of the molecular classification of diffuse large B-cell lymphoma by immunohistochemistry using a tissue microarray. Blood. 2004;103(1):275-82.

5. Cheson BD, Fisher RI, Barrington SF, Cavalli F, Schwartz LH, Zucca E, et al. Recommendations for Initial Evaluation, Staging, and Response Assessment of Hodgkin and Non-Hodgkin Lymphoma: The Lugano Classification. Journal of Clinical Oncology. 2014;32(27):3059-67.

6. Koboldt DC, Zhang Q, Larson DE, Shen D, McLellan MD, Lin L, et al. VarScan 2: somatic mutation and copy number alteration discovery in cancer by exome sequencing. Genome research. 2012;22(3):568-76.

7. Scherer F, Kurtz DM, Newman AM, Stehr H, Craig AF, Esfahani MS, et al. Distinct biological subtypes and patterns of genome evolution in lymphoma revealed by circulating tumor DNA. Sci Transl Med. 2016;8(364):364ra155.
